# Ethnic identity and folk-cultural adaptation: the roles of social distance and social support

**DOI:** 10.3389/fpsyg.2025.1588684

**Published:** 2025-08-08

**Authors:** Ri Hai, Wei Zong, Fang Luo, Linlin Yang, Xiaoming Bai, Yinbu Suo

**Affiliations:** ^1^School of Ethnology and Sociology, Minzu University of China, Beijing, China; ^2^Southwest Frontier Minority Research Center, Yunnan University, Kunming, China; ^3^Kunming Frontier Information Research Center, Kunming, China; ^4^College of Humanities, Honghe University, Mengzi, China; ^5^Institute of Chinese Northern Borderland Culture, Inner Mongolia Academy of Social Sciences, Hohhot, China

**Keywords:** ethnic identity, cultural adaptation, folk culture, social distance, social support

## Abstract

Multinational states typically face dual challenges arising from ethnic identity and folk-cultural adaptation. Balancing these elements is crucial in social science research. While previous studies have highlighted the significant impact of ethnic identity on cultural adaptation, the underlying mechanisms remain underexplored. To address this gap, we conducted a cross-sectional survey of 372 residents in the multi-ethnic border regions of Yunnan, China, during July and August 2024. Using the Ethnic Identity Scale, Social Distance Questionnaire, Social Support Scale, and Revised Sociocultural Adaptation Scale, we measured ethnic identity, social distance, social support, and folk-cultural adaptation, respectively. Our results revealed a positive association between ethnic identity and folk-cultural adaptation, partially mediated by social distance. Additionally, social support moderated both the direct relationship between social distance and folk-cultural adaptation and the indirect effect of ethnic identity on folk-cultural adaptation (via social distance). These findings deepen our understanding of how ethnic identity influences folk-cultural adaptation in multi-ethnic regions, underscoring its role in strengthening ethnic solidarity, promoting cultural diversity, and fostering social cohesion.

## 1 Introduction

In the era of globalization and cultural diversity, ethnic identity and cultural adaptation have become prominent research topics (Arli et al., 2023; Munoz, 2022; Kunuroglu, 2021; Lou, 2021). For nations with multiple ethnic groups, balancing ethnic identity and cultural adaptation is crucial for social harmony and a key focus of social science research (Denisova et al., 2024; Kuang et al., 2023; Bjerre, 2022; Niang, 2022). Thus, exploring the intricate link between them and their underlying mechanisms is both urgent and significant. Culture, first systematically defined by Tylor (1913) as a complex whole comprising knowledge, beliefs, art, law, and morals, is broad and all-encompassing. Folk culture, a key part of culture, is the specific expression of culture in civilian society. It is formed and passed down over history with uniqueness and stability (Zhong, 1998). Folk culture takes diverse forms and shares features like locality, popularity, relative independence, spontaneity, high group identity, and homogeneity (Dundes, 1990). Macro-level research on ethnic identity and cultural adaptation is well-developed, but micro-level studies on ethnic identity and folk-culture adaptation are relatively scarce. Hence, this study focuses on the impact of ethnic identity on folk-culture adaptation from a micro perspective.

When exploring how ethnic identity impacts cultural adaptation, the roles of social distance and social support are key yet often overlooked (Chen and Zhou, 2003). Social distance, a measure of the psychological and emotional gaps between groups (Bogardus, 1925), often weakens cultural adaptation (Otlu, 2010; Redmond, 2000). Social support, which includes emotional, material, and informational assistance (Cohen and Wills, 1985), can reduce this negative impact (Bolaji et al., 2024; English et al., 2021; Mao and Liu, 2016). However, few studies have examined these two variables within the relationship between ethnic identity and cultural adaptation. This study addresses this gap by focusing on the effect of ethnic identity on folk-cultural adaptation among residents in Yunnan Province’s border areas from a micro-perspective. It also delves into the underlying mechanisms of this effect. Specifically, the study looks into the mediating and moderating roles of social distance and social support in the link between ethnic identity and folk-cultural adaptation. Finally, the study proposes an integrated framework that combines ethnic identity, social distance, folk-cultural adaptation, and social support. This framework underscores how social distance and folk-cultural adaptation explain the impact of ethnic identity on folk-cultural adaptation.

## 2 Literature review and hypotheses

### 2.1 Ethnic identity and cultural adaptation

Smith (2000) has noted that the term “ethnic identity” is a central ideal of nationalist ideology and also an analytical concept. Smith defines it as “the continuous reproduction and reinterpretation of the values, symbolic memories, myths, and traditional patterns that constitute the distinctive heritage of a nation, as well as the continuous reinterpretation of personal identity associated with that pattern and heritage and its cultural components.” In addition, Smith (2000) believes that ethnic identity combines two dimensions: one is civic and territorial, and the other is ethnic and blood. In each specific case, the proportion of these two dimensions is different, and in different periods, the two dimensions can be transformed into each other. In general, national identity is the main form of collective identity. No matter how individuals feel, it provides the dominant standard of culture and identity, the fundamental principle of political rule, and the primary focus of social and economic behavior. Today, national identity is not only global but also ubiquitous. Although its importance varies in different contexts, it has permeated the lives of individuals and communities in most areas of activity (Lai and Lin, 2019) In this study, folk-cultural adaptation is conceptualized as a micro-level perspective of cultural adaptation. It refers to the dynamic process where individuals or groups, when accepting and integrating into a specific folk culture, actively or passively adjust and change to better integrate with the cultural environment, interact with it, and maintain their social functions.

According to the Dual Process Model of Acculturation (Berry, 1990), acculturation strategies are divided into two dimensions: one is the maintenance or rejection of an individual’s original culture, and the other is the acceptance or rejection of the host culture. Based on these two dimensions, Berry proposed four acculturation strategies: integration, assimilation, separation, and marginalization. Ethnic identity belongs to the first dimension; when individuals have a strong sense of ethnic identity, they are more likely to actively maintain and pass on their own folk culture. This positive ethnic identity helps individuals adopt the “integration” strategy when engaging with other cultures, that is, to maintain their own culture while actively adapting to other cultures. Scholars generally agree that the stronger an individual’s ethnic identity, the greater their capacity for cultural adaptation (Gusman et al., 2024; Ngo and Li, 2016; Cleveland et al., 2009). For example, empirical research by Pektas et al. (2023) shows that ethnic identity is positively correlated with in-group ethnic-racial socialization, and ethnic identity is also positively correlated with positive out-group ethnic-racial socialization. Based on the above academic views, this paper starts from the micro level and believes that national identity has a significant influence on the adaptation of folk culture to residents in multi-ethnic areas. The “integration” strategy avoids both the separation caused by over-adherence to one’s own culture and the assimilation or marginalization caused by completely abandoning one’s own culture (van Bergen et al., 2019). Therefore, this paper proposes the following hypothesis:

*Hypothesis 1*: Ethnic identity is positively associated with folk-cultural adaptation.

### 2.2 The mediating effect of social distance

The concept of social distance was first introduced by the German sociologist Simmel (2009), who defined it as “the degree of closeness or distance between individuals or groups in psychological, emotional, or social interactions.” Simmel regarded social distance as an “internal barrier” between people, emphasizing its subjective nature. Subsequently, the American sociologist Park (1924), of the Chicago School, further developed this concept, defining it as the level of intimacy between individuals or groups and noting that this distance could be measured through social interaction. Furthermore, by understanding social distance, we can better explain intergroup prejudice, discrimination, and conflict and also provide a theoretical foundation for promoting social harmony and group integration (Preiss et al., 2023). In multi-ethnic nations, reducing social distance between ethnic groups can be achieved through increased contact, cooperation, and the pursuit of shared goals, thereby promoting ethnic unity and social stability (Williamson et al., 2024).

Gaertner et al. (1993) proposed the Common Ingroup Identity Model, which suggests that by recategorizing members of two originally distinct groups into a larger common group, the cognitive representation of group members can be altered. This recategorization reduces the sense of boundary between groups, enhances members’ identification with the common group, and thereby diminishes intergroup bias. When different ethnic groups identify with a common superordinate group, such as the Chinese nation, the social distance between groups is significantly shortened. This shared identification reduces prejudice and discrimination between groups and increases group intimacy, meaning that stronger ethnic identity is associated with shorter social distance. Most scholars also generally agree that ethnic identity is a precursor variable of social distance and has a negative impact on individuals’ social distance. For example, Yin et al.'s (2019) experimental study shows that ethnic identity has a significant negative impact on social distance.

Allport et al. (1954), Intergroup Contact Theory emphasizes that increasing contact between different groups can reduce prejudice and discrimination and enhance understanding and acceptance between groups. In the context of ethnic identity and folk-cultural adaptation, the reduction of social distance provides opportunities for contact between different ethnic groups, thereby promoting folk-cultural adaptation. It is evident that social distance is a significant factor influencing folk-cultural adaptation. The greater the social distance, the greater the challenges individuals or groups face when adapting to a new cultural environment. Previous studies have further indicated that there is a significant negative correlation between individuals’ sense of social distance and their capacity for folk-cultural adaptation. For instance, to better understand the cultural adaptation process, Yan et al. (2024) employed a cross-lagged panel model with a longitudinal approach to capture the structural relationships between variables over time, demonstrating that higher levels of perceived cultural distance lead to lower social and cultural adaptation abilities.

To sum up, on the one hand, the enhancement of ethnic identity helps to reduce social distance; on the other hand, the reduction of social distance signifies an increase in individuals’ capacity for folk-cultural adaptation. As Fedotova (2021) described in her empirical study based on 248 students, students from Arab countries possess strong ethnic identities and take pride in their ethnic groups. When adapting to a foreign cultural environment, they are endowed with both integration and assimilation strategies for cultural adaptation. In addition, cultural distance affects the social and cultural adaptation of students from Arab countries studying in Russia, with a high degree of cultural distance leading to poor social and cultural adaptation. Therefore, based on the views of the aforementioned scholars, this paper argues that social distance plays a mediating role between ethnic identity and folk-cultural adaptation among residents in multi-ethnic regions; that is, ethnic identity indirectly affects their capacity for folk-cultural adaptation through social distance. Thus, the following research hypotheses are proposed:

*Hypothesis 2*: Social distance mediates the association between ethnic identity and folk-cultural adaptation.

### 2.3 The moderating effect of social support

Cohen and Wills (1985) noted that social support refers to the beneficial interpersonal interactions that protect individuals from the adverse effects of stressful events. It serves as a cognitive evaluation of the closeness and quality of an individual’s interpersonal relationships and is a crucial factor in adapting to various social environments. Cullen (1994), on the other hand, approached social support from the perspective of its functions, viewing it as the material or psychological assistance that individuals receive from their communities, social networks, or relatives and friends. Furthermore, the Social Support Theory (Cohen and Wills, 1985) posits that social support can alleviate the difficulties individuals face when dealing with stress and adapting to new environments by providing emotional, informational, and instrumental help, thereby promoting psychological and behavioral adaptation. This theory has been widely applied in multiple fields, including cross-cultural adaptation (Zhang et al., 2024), mental health (Yang et al., 2023; Gan et al., 2025; Hefner and Eisenberg, 2009), and social isolation (Jun et al., 2024). Previous studies have also further demonstrated that social support is an important predictor of cultural adaptation (Luh et al., 2024; Zheng and Ishii, 2023). In other words, the perceived social support of individuals is conducive to enhancing their capacity for cultural adaptation. For example, an empirical study by Wu et al. (2024), based on 1,108 mainland Chinese college students, showed that self-esteem indirectly influences college students’ sociocultural adaptation abilities through the consecutive mediating effects of social support and school belongingness.

Given the significant impact of social distance on cultural adaptation, from the micro level, its interaction with social support may affect the cultural adaptation of folk in multi-ethnic areas. According to the Stress Buffering Model (Cohen and Wills, 1985), social support, as a buffering mechanism, can mitigate the stress that residents in multi-ethnic regions encounter during the process of cultural adaptation, thereby promoting folk-cultural adaptation. In other words, social support helps individuals to alleviate cultural conflicts and psychological stress by providing emotional and practical assistance, thereby reducing social distance. Specifically, social support can buffer the negative impact of social distance on cultural adaptation and help individuals better adapt to new cultural environments (Hinz et al., 2025; English et al., 2021; Waldron et al., 2005). Previous research has also further demonstrated that social support plays a moderating role in the correlation between social distance and cultural adaptation (Fan et al., 2012; Luo et al., 2024). For example, a study by Ng et al. (2017) based on 188 mainland Chinese college students staying in Hong Kong showed that social support from family members, local peers, and non-local peers heightens the positive impact of integration strategies and buffers the negative impact of marginalization strategies on sociocultural and psychological adaptation. On the basis of these arguments, we hypothesize that residents who perceive adequate social support will exhibit lower social distance, thereby facilitating better adaptation to the local folklore culture, as stated in Hypothesis 3.

*Hypothesis 3:* The association between social distance and folk-cultural adaptation is moderated by social support.

Based on the above-mentioned findings on moderation, we also anticipated that the mediation of social distance in the relationship between ethnic identity and folk-cultural adaptation would be moderated by social support. Specifically, a high level of perceived social support among residents in multi-ethnic regions may enhance the impact of ethnic identity on folk-cultural adaptation by reducing social distance. In other words, when residents in multi-ethnic regions perceive substantial social support from family members, relatives, and friends, they are likely to experience reduced social distance, thereby facilitating their folk-cultural adaptation. Conversely, when such residents perceive a lack of social support, they are more likely to experience increased social distance and less effective folk-cultural adaptation. Following this reasoning, we expected the indirect effects of ethnic identity on folk-cultural adaptation through social distance to vary depending on the level of social support. We therefore posit the following:

*Hypothesis 4:* The mediation effect of ethnic identity on folk-cultural adaptation through social distance is moderated by social support.

**Figure 1:**
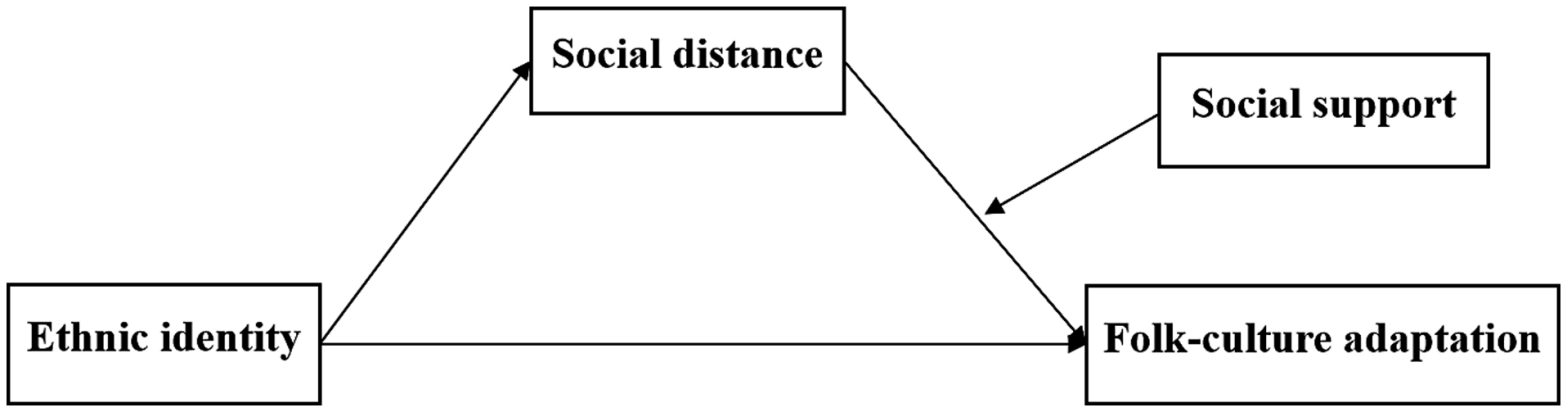
Hypothesized research model.

The research model is presented in Figure 1.

## 3 Methods

### 3.1 Data and sample

To ensure the scientific rigor and accuracy of this study, the researchers employed a targeted sampling method to ensure that the survey sample adequately represents residents from multi-ethnic border regions of Yunnan Province. Initially, researchers used cluster sampling to identify four prefectures (cities) as the main investigation areas from eight prefectures (cities) along the Yunnan border: Lincang City, Nujiang Lisu Autonomous Prefecture, Dehong Dai and Jingpo Autonomous Prefecture, and Baoshan City. Subsequently, four representative investigation sites were determined through random sampling: Fengqing County, Lincang City; Liuku Street, Lushui City, Nujiang Lisu Autonomous Prefecture; Ruili City, Dehong Dai and Jingpo Autonomous Prefecture; and Longyang District, Baoshan City. Before the formal investigation began, four undergraduate students were recruited by the researchers, who then underwent training in investigation methods and data collection. One administrative village was randomly selected from each of the four investigation sites for field investigation. Finally, between July 15, 2024, and August 30, 2024, researchers and investigators conducted data collection in the multi-ethnic regions of Yunnan Province, including Lincang, Nujiang, Dehong, and Baoshan.

We adopted a combined approach of offline recruitment and online survey completion to collect data. During the survey process, participants were asked to complete the questionnaires on a voluntary and anonymous basis and were informed that they could discontinue the survey at any time if they felt uncomfortable with any of the content. Ultimately, after obtaining written informed consent from the participants, we distributed 400 questionnaires and collected 390. After excluding incomplete and invalid ones, 372 valid questionnaires were obtained, representing an effective return rate of 93%. Of all participants, 190 were male (51.08%) and 182 were female (48.92%). In terms of age distribution, 71.5% of participants were aged 18–30 years, while 28.5% were over 30, with no minors included. Regarding educational background, 56.5% of participants held at least a bachelor’s degree. In terms of ethnicity, 107 participants were Han Chinese (28.76%), and 265 were from ethnic minorities (71.24%). As for place of residence, 105 participants lived in Nujiang, Yunnan (28.23%); 85 in Lincang, Yunnan (22.85%); 79 in Dehong, Yunnan (21.24%); and 103 in Baoshan, Yunnan (27.68%).

### 3.2 Measurements

In this study, we recognize that the scales used, classic Western instruments, may have cross-cultural differences. To better adapt them to the residents in Yunnan’s border areas and minimize survey errors, we translated and back-translated the scales per cross-cultural survey standards to ensure semantic consistency across cultures. Translation involved local culture- and language-savvy professionals to retain the original meaning and suit local cultural expression. Also, before the formal survey, a pilot test in Yunnan’s multiethnic areas was done to evaluate scale applicability and validity. Via the pilot test, we refined and optimized scale items for better local-culture adaptation and improved validity and reliability.

#### 3.2.1 Predictor variable: ethnic identity

The variable of ethnic identity was investigated using the 20-item scale developed by Phinney (1992). An example item is “I actively participate in organizations or social groups that are primarily composed of members of my own ethnic group.” Each item was scored on a 5-point Likert scale, with 1 indicating “strongly disagree” and 5 indicating “strongly agree.” In this study, the Cronbach’s *α* coefficient for this scale was 0.865.

#### 3.2.2 Outcome variable: folk-cultural adaptation

The variable of folk-cultural adaptation was measured using a scale adapted from the Revised Sociocultural Adaptation Scale (SCAS-R) developed by Wilson (2013), which consists of 15 items. An example item is “I try to adapt to the lifestyle of the mainstream culture while also maintaining some traditions of my own ethnic group.” Each item was scored on a 5-point Likert scale, with 1 indicating “strongly disagree” and 5 indicating “strongly agree.” In this study, the Cronbach’s *α* coefficient for this scale was 0.804.

#### 3.2.3 Mediating variable: social distance

The variable of social distance was measured using a questionnaire adapted from the Social Distance Scale developed by Bogardus (1925). The questionnaire employs a 1–5 point scale, ranging from “very unwilling” to “very willing,” and consists of four items, such as “Would you be willing to work with people from other ethnic groups?” Higher scores indicate a weaker sense of social distance. In this study, the Cronbach’s α coefficient for this scale was 0.943. In addition, we conducted a confirmatory factor analysis on the adapted scale, and the results showed that the scale *χ*^2^/DF = 0.577, RMR = 0.002, RMSEA = 0.000, and all the indexes of model fitting were within the ideal standard range, indicating that the model fitting was good.

#### 3.2.4 Moderator variable: social support

Social support was measured using the 12-item scale developed by Zimet et al. (1988), which has been widely used in research across diverse cultural contexts and is well-established and widely applicable (Yang et al., 2023). An example item from the scale is “My friends can provide a lot of help.” Each item was scored on a 5-point Likert scale, with 1 indicating “strongly disagree” and 5 indicating “strongly agree.” In this study, the Cronbach’s α coefficient for this scale was 0.950.

#### 3.2.5 Control variables

In this study, considering the impact of demographic factors on cultural adaptation among residents in multi-ethnic regions, we selected gender, age, annual income, and social fairness as control variables. Income, measured as the average annual income over the past 5 years, reflects an individual’s economic status and living resources, which can influence their attitudes toward folk culture and their adaptive behaviors. Additionally, social fairness reflects an individual’s perception of overall social equity. During the process of cultural adaptation, if individuals feel a sense of social fairness, they may believe that they will receive equal opportunities and respect during cultural interactions and adaptation, thereby enhancing their positive feelings about ethnic identity and becoming more proactive in adapting to folk culture.

### 3.3 Data processing and analysis

In this study, we employed a three-step analytical approach for data processing and analysis. First, using the SPSS 27.0 statistical software package, we performed expectation–maximization (EM) imputation to handle missing data and conducted descriptive statistics, Pearson correlation analysis, and common method bias tests of the variables. Second, we constructed a structural equation model (SEM) using the Process macro (Model 4) with bootstrapping to examine the mediating role of social distance in the relationship between ethnic identity and folk-cultural adaptation among residents in multi-ethnic regions. Finally, we utilized the PROCESS macro (Model 14) to verify the moderating effect of social support.

## 4 Results

### 4.1 Descriptive statistics and correlations

The descriptive, correlation, and reliability results regarding the variables of interest are presented in Table 1. Ethnic identity was negatively associated with social distance (r = −0.35, *p* < 0.010), but it was positively related to folk-cultural adaptation (r = 0.63, *p* < 0.010) and social support (r = 0.51, *p* < 0.010). Social distance was negatively correlated with folk-cultural adaptation (r = −0.46, *p* < 0.010) and social support (r = −0.10, *p* < 0.050). Finally, social support was positively associated with folk-cultural adaptation (r = 0.26, *p* < 0.010).

**Table 1:** Means, standard deviations, and correlations among variables.

Variables	1	2	3	4	5	6	7	8
Gender	1							
2. Age	−0.20**	1						
3. Annual income	−0.13**	0.47**	1					
4. Social fairness	−0.08	0.08	0.12*	1				
5. Ethnic identity	−0.06	−0.02	0.10	0.23**	(0.865)			
6. Social distance	−0.11*	0.08	−0.12*	−0.07	−0.35**	(0.943)		
7. Social support	0.06	−0.01	0.12*	0.20**	0.51**	−0.46**	(0.950)	
8. Folk-cultural adaptation	−0.14**	0.07	0.12*	0.19**	0.63**	−0.10*	0.26**	(0.804)
Mean	1.49	28.02	4.88	3.25	3.59	1.80	3.87	3.35
SD	0.50	8.62	2.94	0.89	0.50	0.74	0.67	0.55

*N* = 372. *p < 0.050, **p < 0.010, and ***p < 0.001. Boldface values indicate Cronbach’s alpha. SD, standard deviation.

### 4.2 Testing for common method bias

Due to single data collection, item characteristics of the scale, and response tendencies of the respondents, common method bias (CMB), a systematic error, is widespread in empirical research (Podsakoff et al., 2003). To mitigate the risk of CMB, several precautions were taken during the survey process, such as ensuring respondent anonymity and using reverse wording for some questions. Additionally, to rigorously assess the potential presence of common method bias, the Harman one-factor test (Calvete et al., 2010) was conducted before analyzing the data. The results indicated that, without rotation, nine factors with eigenvalues >1 were extracted, explaining 67.088% of the total variance. The first factor accounted for 29.424% of the variance, which is below the 40% threshold, suggesting that common method bias was not a significant issue. Thus, the data were deemed valid for analysis, with common method bias not posing a significant threat. The Cronbach’s alpha values of all the scales exceeded 0.70, indicating good inter-item reliability.

### 4.3 Hypothesis testing

#### 4.3.1 Mediating effect analysis of social distance

In this study, we first standardized the variables. Then, using Model 4 in PROCESS, we drew 5,000 bootstrap samples. Controlling for gender, age, annual income, and social fairness, we analyzed the mediating effect of social distance between ethnic identity (independent variable) and folk-cultural adaptation (dependent variable). For the specific analysis results, refer to Table 2.

**Table 2:** Results of mediating hypotheses.

Variables	Social distance	Folk-cultural adaptation	
	Model 1	Model 2	Model 3
Constant	3.73(0.33) ***	0.91(0.20)***	0.59(0.23)*
Gender	−0.18(0.07)*	−0.09(0.04)*	−0.08(0.04)
Age	0.01(0.00)*	0.00(0.00)	0.00(0.00)
Annual income	−0.04(0.01)**	0.00(0.01)	0.01(0.01)
Social fairness	0.00(0.04)	0.03(0.03)	0.03(0.03)
Ethnic identity	−0.49(0.07) ***	0.66(0.04)***	0.71(0.05)***
Social distance			0.09(0.03)**
Total effect [95% CI]		0.66 [0.57, 0.75]	
Direct effect [95% CI]		0.71 [0.61, 0.80]	
Indirect effect [95% CI]		−0.04 [−0.09, −0.01]	
R^2^	0.16***	0.41***	0.42***

Bootstrap size = 5,000. CI, confidence interval. *p < 0.050, **p < 0.010, and ***p < 0.001.

Model 1 shows that ethnic identity significantly and negatively predicts social distance (B = −0.49, *p* < 0.001). Model 2 indicates that ethnic identity significantly and negatively predicts folk-cultural adaptation (B = 0.66, *p* < 0.001). Model 3 reveals that social distance significantly and positively predicts folk-cultural adaptation (B = 0.09, *p* < 0.001). Moreover, the bootstrap-derived indirect effect of ethnic identity on folk-cultural adaptation was significant (B = −0.04, 95% CI: [−0.09, −0.01]). Since the 95% CI for the indirect effect does not include zero, the mediating effect is significant. Thus, social distance partially mediates the relationship between ethnic identity and folk-cultural adaptation.

#### 4.3.2 Moderating effect of social support

In this study, we first standardized the variables. Using Model 14 in PROCESS, we drew 5,000 bootstrap samples. Controlling for gender, age, annual income, and social fairness, we analyzed the mediated moderation effect of social support on the relationship between ethnic identity (independent variable), social distance (mediator), and folk-cultural adaptation (dependent variable). The interaction between social distance and social support has a significant positive effect on the folk-cultural adaptation of residents in multi-ethnic regions (B = 0.13, *p* < 0.001; Table 3).

**Table 3:** Results of moderating hypotheses.

Variables	B	SE	*p*	Boot LLCI	Boot ULCI
Outcome variables: folk-cultural adaptation					
Constant	1.52	0.32	0.000	0.90	2.15
Gender	−0.09	0.04	0.051	−0.17	0.00
Age	0.00	0.00	0.531	−0.00	0.01
Annual income	0.01	0.01	0.306	−0.01	0.03
Social fairness	0.02	0.02	0.366	−0.03	0.07
Ethnic identity (X)	0.72	0.05	0.000	0.62	0.82
Social distance (M)	−0.37	0.11	0.001	−0.59	−0.15
Social support (W)	−0.26	0.07	0.000	−0.39	−0.13
M × W	0.13	0.03	0.000	0.07	0.19
R^2^ = 0.45***, *F*-value = 37.01					

Bootstrap size = 5,000. Boot SE, bootstrapping standard error; LL, low limit; UL, upper limit; CI, confidence interval.

**Figure 2:**
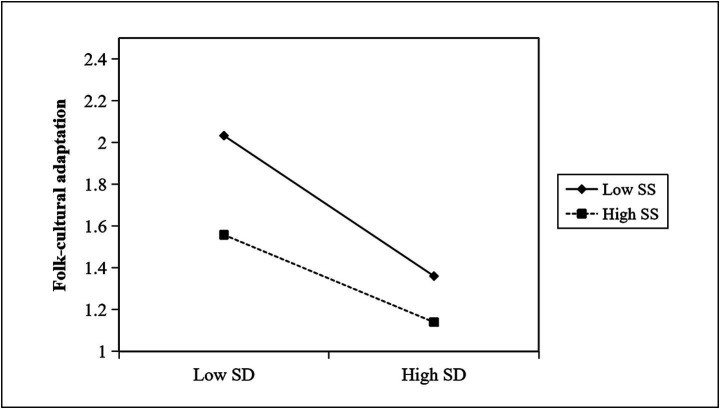
The moderating effect of social support.

We constructed a social support moderation effect diagram (Figure 2) to further explain the results. The slope is −0.67 when social support is at a low level and −0.41 when social support is at a high level. Figure 2 shows that the effect of social distance on folk-cultural adaptation was weaker among those residents who perceived higher levels of social support.

Finally, to test the hypothesized moderated mediation, we determined the conditional indirect effect of ethnic identity on folk-cultural adaptation through social distance at the mean level of social support, one standard deviation above the mean, and one standard deviation below the mean. Table 4 shows that when the perceived level of social support among residents in multi-ethnic regions is at a medium (B = −0.06, 95% CI: [−0.11, −0.02]) or higher level (B = −0.10, 95% CI: [−0.18, −0.04]), ethnic identity exerts a significant indirect effect on folk-cultural adaptation through social support. Conversely, when the perceived level of social support is low (B = −0.02, 95% CI: [−0.06, 0.01]), this indirect effect is non-significant (the bootstrap confidence interval includes zero). Overall, the index of moderated mediation is significant (B = −0.06, 95% CI: [−0.10, −0.02]).

**Table 4:** Results for the index of moderated mediation and conditional indirect effects.

Moderator: social support	Effect/Index	Boot SE	Boot LLCI	Boot ULCI
Conditional indirect effects [95% CI]				
M - SD (−0.67)	−0.02	0.02	−0.06	0.01
Mean	−0.06	0.02	−0.11	−0.02
M + SD (+0.67)	−0.10	0.03	−0.18	−0.04
Index of moderated mediation [95% CI]				
Social support	−0.06	0.02	−0.10	−0.02

Bootstrap size = 5,000. Boot SE, bootstrapping standardized error; LL, low limit; UL, upper limit; CI, confidence interval. *p < 0.05, **p < 0.01, and ***p < 0.001.

## 5 Discussion and conclusion

The research results indicate a significant positive correlation between ethnic identity and folk-cultural adaptation. Social distance partially mediates this relationship, and social support can lessen the impact of social distance on folk cultural identity.

The findings align with Hypothesis 1, showing a significant positive correlation between ethnic identity and folk-cultural adaptation, which is consistent with prior studies (Gusman et al., 2024; Ngo and Li, 2016). Drawing from the Dual Process Model of Acculturation, this study delves into the intricate relationship between ethnic identity and cultural adaptation. The model emphasizes the bidirectional nature of acculturation, where individuals can maintain their unique cultural identity while actively engaging with the dominant culture. In the context of multiethnic regions, a strong ethnic identity not only instills a sense of pride and belonging but also equips individuals with the cultural resources and confidence needed to navigate and adapt to the broader cultural landscape. This dual process allows individuals to bridge their own cultural heritage with the cultural practices of the wider community, thereby fostering a more harmonious and inclusive social environment. Moreover, the positive correlation between ethnic identity and folk-cultural adaptation can be further understood through the lens of social identity theory. A robust ethnic identity provides individuals with a clear sense of self and a positive social identity, which in turn motivates them to actively participate in and contribute to the cultural vitality of their community. This active participation reinforces their connection to their cultural roots while enabling them to adapt to and appreciate other cultural traditions within the multiethnic society. Thus, our research not only corroborates existing findings but also enriches the theoretical dialogue on how ethnic identity serves as a cornerstone for cultural adaptation and integration in diverse settings, offering valuable insights for fostering cultural cohesion and understanding in multiethnic regions (Pektas et al., 2023).

Consistent with hypothesis 2, social distance partially mediates the link between ethnic identity and folk-cultural adaptation, which is similar to previous research results (Deng et al., 2024; Tang et al., 2024). From the perspective of the Common Ingroup Identity Model and Intergroup Contact Theory, ethnic identity can reduce social distance by promoting cross-group interaction and the formation of a common ingroup identity. The reduction of social distance further fosters more positive attitudes and behaviors toward folk-cultural adaptation. This process reflects an integrated view of the two theories in explaining the mediating role of social distance between ethnic identity and folk-cultural adaptation. More specifically, the Common Ingroup Identity Model suggests that individuals are more likely to have positive attitudes and behaviors toward others when they perceive themselves and others as part of a larger common ingroup. Strong ethnic identity encourages individuals to engage in cross-ethnic interactions, which can lead to the formation of a broader common ingroup identity that encompasses multiple ethnic groups. As a result, the social distance between different ethnic groups is reduced. On the other hand, Intergroup Contact Theory posits that positive contact between different groups can reduce prejudice and stereotypes and enhance mutual understanding and tolerance. When individuals with strong ethnic identities actively participate in intergroup contact, they gain a better understanding of other ethnic cultures, which in turn reduces social distance and promotes folk-cultural adaptation. Therefore, this study further strengthens the empirical evidence for the partial mediating role of social distance in the relationship between ethnic identity and folk-cultural adaptation.

Consistent with Hypotheses 3 and 4, social support moderates the relationship between social distance and folk-cultural adaptation and also moderates the mediation effect of ethnic identity on folk-cultural adaptation through social distance. This is in line with prior studies (Fan et al., 2012; Luo et al., 2024). The stress buffering model highlights that social support lessens the negative impact of stressors, such as social distance, which can cause feelings of isolation and non-acceptance during folk-cultural adaptation. When individuals have strong social support from family, friends, or the community, they receive emotional comfort, practical help, and coping strategies. This enables them to better handle the stress from social distance and adapt to folk culture more effectively. In addition, the model also clarifies that the indirect effect of ethnic identity on folk-cultural adaptation through social distance is moderated by social support. On one hand, strong ethnic identity may prompt individuals to actively engage in cross-ethnic interactions, reduce social distance, and enhance folk-cultural adaptation. On the other hand, even if there is still some social distance, ample social support can enhance individuals’ ability to overcome difficulties and persist in the adaptation process. Thus, social support plays a buffering role in the relationship between social distance and folk-cultural adaptation, as well as in the indirect effect of ethnic identity on folk-cultural adaptation through social distance. Overall, our study strengthens the empirical evidence for social support as a moderator.

### 5.1 Theoretical implications

This study delves into the intricate relationship between ethnic identity and folk-cultural adaptation by incorporating social distance and social support as mediators and moderators. It provides a rich theoretical framework that helps elucidate the multifaceted mechanisms of cultural adaptation within multiethnic contexts. While the study does make a theoretical contribution by integrating these variables into the existing models of cultural adaptation, it is important to acknowledge that the theoretical innovation could be further strengthened by incorporating more nuanced conceptual insights or by more thoroughly integrating with established theories such as Berry’s acculturation framework or sociocultural adaptation theory. Additionally, future research could explore alternative interpretations and a broader range of contextual variables to enhance the depth and comprehensiveness of the theoretical framework.

Moreover, this study goes further to confirm via empirical research that social distance acts as a mediator between ethnic identity and folk-cultural adaptation, while social support plays a moderating role. These findings not only provide empirical validation but also extend the application contexts of the Dual Process Model of Acculturation and the Stress Buffering Model. By doing so, the study offers new theoretical insights and perspectives that can guide future research in related fields. The identification of these mechanisms contributes to a deeper understanding of how cultural adaptation processes function in multiethnic settings, highlighting the interplay between individual psychological constructs and social dynamics. Furthermore, the study’s findings imply that by enhancing social support and reducing social distance, more effective interventions can be developed to promote cultural adaptation. This not only benefits individuals but also fosters greater social cohesion within diverse communities. The integration of these variables into existing theoretical frameworks strengthens the explanatory power of the models and provides a more nuanced lens through which to view the complexities of cultural adaptation. As such, this study makes a meaningful theoretical contribution to the broader discourse on acculturation and intergroup relations.

Furthermore, this study delves into the positive role of ethnic identity in cultural adaptation within multi - ethnic regions. It highlights the bidirectional dynamic relationship between preserving one’s unique cultural heritage and integrating into the mainstream culture. This dual process is crucial for constructing a harmonious and inclusive multicultural theory. By maintaining cultural uniqueness, individuals can strengthen their sense of identity and belonging, which in turn provides them with the confidence and resources to actively engage with and adapt to the mainstream culture. Conversely, successful integration into the broader cultural context can enhance individuals’ appreciation for their own cultural background and foster a more positive attitude toward cultural diversity. The study’s emphasis on this bidirectional relationship offers a more nuanced understanding of cultural adaptation, moving beyond simplistic notions of assimilation or segregation. Instead, it presents a model where cultural preservation and cultural integration can coexist and reinforce each other, paving the way for the development of more comprehensive and inclusive multicultural theories that can better address the complexities of modern multi-ethnic societies.

In addition, the findings shed light on the crucial role of social support in reducing the adverse effects of social distance. This discovery holds significant theoretical implications for social policymakers and community workers. By understanding this relationship, they can develop more effective cultural adaptation interventions. Such interventions are not only instrumental in helping individuals overcome the challenges posed by social distance but also in fostering a greater sense of belonging and inclusion within diverse communities. These initiatives can take various forms, such as communitybased programs that encourage intercultural interactions, support networks that provide practical and emotional assistance, and policies that promote social inclusion and reduce discrimination. By implementing these strategies, it becomes possible to enhance social cohesion, enabling individuals from different ethnic backgrounds to coexist harmoniously. Furthermore, promoting cultural consensus becomes more achievable as people develop a deeper appreciation and respect for each other’s cultural practices and values. In multiethnic societies, where cultural diversity is both a richness and a challenge, these outcomes are vital for building stronger, more unified communities that can thrive on their diversity rather than being divided by it. The study’s emphasis on the practical applications of its findings bridges the gap between theory and practice, making it a valuable resource for those working toward social harmony and cultural integration.

### 5.2 Practical implications

First, the government should integrate the construction of social support networks into the governance framework of multi-ethnic regions, reducing structural social distance through economic policies and culturally inclusive measures. The Common Ingroup Identity Model (Gaertner et al., 1993) can be adopted to integrate multiculturalism into a higher-level collective identity. Additionally, mixed housing policies should be implemented to mitigate the reinforcing effect of residential segregation on psychological alienation (Preiss et al., 2023), and support for ethnic cultural and special industries can promote cross-group economic cooperation (Cleveland et al., 2009).

Secondly, the education system needs to methodically incorporate the development of cultural adaptability. Research underscores that in multi-ethnic regions, ethnic characteristics hold substantial importance, and ethnic identity significantly impacts cultural adaptability. Existing studies also indicate that integrating ethnic history with personal experiences can enhance the understanding of identity dynamics (Phinney, 1992). Thus, this study suggests that government departments and educational institutions should work together to embed ethnic cultural education into school curricula, thereby assisting students in reducing cultural prejudices.

Thirdly, the healthcare system can also benefit from the findings of this study. In multi-ethnic regions, cultural adaptability and social support are crucial factors that influence the utilization and effectiveness of healthcare services. By understanding the mediating role of social distance and the moderating effect of social support on folk-cultural adaptation, healthcare providers can develop more culturally sensitive and inclusive practices. For example, community health interventions that foster social support networks can mitigate the negative effects of social distancing, thereby enhancing health outcomes. This approach aligns with the broader goal of addressing health disparities in multi-ethnic societies and promoting health equity.

Finally, managers of multi-ethnic regions should fully utilize the adaptive capacity of digital technology to strengthen cultural adaptability within community contexts. The results of this study show that social distance and social support significantly predict residents’ levels of cultural adaptability, which may provide theoretical support for regional cultural integration. Digital technology can serve as a tool to reduce social distance through online and offline integration, playing an integrative role in community culture.

### 5.3 Limitations and future research directions

Like any study, this research also has certain limitations that should be considered in future work. First, the cross-sectional nature of the data limits our ability to establish causal relationships between variables. Although we have examined the relationships among ethnic identity, social distance, folk-cultural adaptation, and the moderating role of social support, longitudinal research is needed to further clarify the causal mechanisms and the dynamic changes over time in the border regions of Yunnan Province.

Second, this study relied on self-reported data, which may be subject to common method bias and social desirability bias. Participants might have provided responses that they believed were socially acceptable or aligned with the researchers’ expectations, potentially distorting the true relationships between variables. Future research could consider using objective measures or multiple data collection methods, such as interviews, observations, and behavioral assessments, to triangulate the findings and reduce the reliance on self-reports.

Lastly, due to the complex linguistic environment of the surveyed villages and the monolingual status of the investigators, the sample was skewed toward younger and more highly educated individuals. Consequently, the demographic data does not fully represent the general population of Yunnan’s multi-ethnic regions, suffering from underrepresentation and generalizability issues. Future research should expand the sampling scope, employ multiple data-collection methods, and leverage big data and AI technologies to address these shortcomings.

## Data Availability

The datasets used in the current study are available from the corresponding author on reasonable request.
